# Bulk-edge correspondence of classical diffusion phenomena

**DOI:** 10.1038/s41598-020-80180-w

**Published:** 2021-01-13

**Authors:** Tsuneya Yoshida, Yasuhiro Hatsugai

**Affiliations:** Department of Physics, University of Tsukuba, Ibaraki, 305-8571 Japan

**Keywords:** Condensed-matter physics, Topological matter

## Abstract

We elucidate that diffusive systems, which are widely found in nature, can be a new platform of the bulk-edge correspondence, a representative topological phenomenon. Using a discretized diffusion equation, we demonstrate the emergence of robust edge states protected by the winding number for one- and two-dimensional systems. These topological edge states can be experimentally accessible by measuring diffusive dynamics at edges. Furthermore, we discover a novel diffusion phenomenon by numerically simulating the distribution of temperatures for a honeycomb lattice system; the temperature field with wavenumber $\pi$ cannot diffuse to the bulk, which is attributed to the complete localization of the edge state.

## Introduction

In these decades, the notion of topology in condensed matter physics enhances its significance^1–7^. One of the characteristic topological phenomena is the emergence of robust gapless edge states due to topological properties in the bulk which is known as the bulk-edge correspondence; the chiral edge states emerge^8^ corresponding to a finite value of the Chern number in the bulk of systems without symmetry^9^, which is elucidated in Ref.^10^. The topologically protected edge states are sources of novel phenomena, such as the quantized Hall conductance^9,11^, the emergence of Majorana fermions^12–18^, etc.

Remarkably, recent works extended the bulk-edge correspondence to several classical systems which are governed by Maxwell equations, Newton equation, etc.^19–35^. These progresses beyond quantum systems provide universal understanding from the topology and result in invention of new devises (e.g., the topological laser^36,37^) thanks to the robust edge states. Therefore, further extending the bulk-edge correspondence beyond quantum systems is considered to be significant in term of both the scientific viewpoint and applications.

In this paper, we point out that classical diffusive systems can be a new platform of the bulk-edge correspondence, which highlights topological aspects of the classical diffusion phenomena; the diffusive systems include a wide variety of systems (e.g., thermal diffusion^38,39^, diffusion of impurities in metals^40^, diffusion of droplets of inks in water, etc.). To this aim, we discretize the diffusion equation based on Fick’s law. The discretized diffusion equation allows us to discuss the bulk-edge correspondence of diffusion phenomena for classical systems; the governing equation is expressed in a matrix form that is mathematically equivalent to a tight-binding model of a quantum system. Our numerical data verify the bulk-edge correspondence for diffusion phenomena in the classical systems. Furthermore, our numerical simulation of the temperature distribution elucidates a novel diffusion phenomenon for a honeycomb lattice system; the temperature field with wavenumber $k_x=\pi$ cannot diffuse into the bulk, which is attributed to the complete localization of the edge state with $k_x=\pi$. Here, we stress that systems we discuss are classical systems in contrast to a previous work^41^ analyzing topology of diffusion of electrons.

## Discretizing the diffusion equation

We introduce a discretized diffusion equation [see e.g., Eq. (3)] based on Fick’s law.

Before addressing the discretization, let us briefly review Fick’s law and the diffusion equation of a continuum scalar field $\phi (t,x)$ in one dimension

1 $$ \partial _t \phi (t,x) = D \partial ^2_x \phi (t,x), $$

where $\partial _{t(x)}$ denotes derivative with respect to time *t* (spatial coordinate *x*). Here, depending on which system we consider, the scalar field $\phi (t,x)$ corresponds to the field of temperatures, the density of the diffusing material, etc.. Fick’s law indicates that the corresponding flux *J* is given by $J=-D\partial _x \phi (t,x)$, where *D* is the diffusion coefficient. By combining this equation and the equation of continuity $\partial _t \phi (t,x)+\partial _x J (t,x)=0$, we obtain the diffusion Eq. (1).

Now, let us discretize the diffusion Eq. (1) connecting the diffusion phenomena to tight-binding models of quantum systems. In order to show the essential idea, we focus on one-dimensional systems.

**Figure 1:**
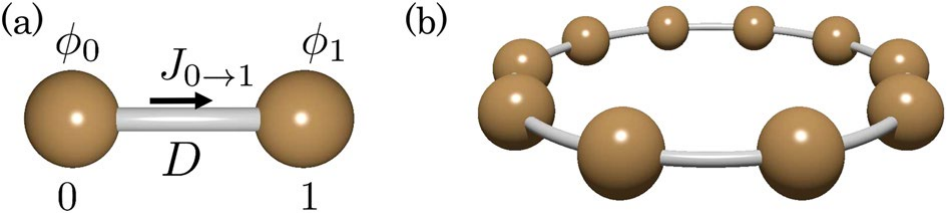
(Color Online). Sketch of the one-dimensional system. (**a**) System composed of two sites coupled with the diffusion coefficient *D*; the flux flowing from site 0 to site 1 is written as $\vec {J}_{0\rightarrow 1}=-D (\phi _0-\phi _1)$, where $\phi$’s denote the discretized field. (**b**) One-dimensional chain under the periodic boundary condition for $L_x=10.$

Consider a system composed of two sites where the values of the discretized field $\phi _{0}$ and $\phi _{1}$ are assigned at each site (see Fig. 1a); for the heat conduction equation, consider two balls (e.g., macroscopic iron balls) where temperatures are $T_0$ and $T_1$. Recalling Fick’s law, we can write the flux flowing from site 0 to 1 with $\phi$’s, $J_{0\rightarrow 1} =-D (\phi _0-\phi _1)$. Here, we have chosen the distance between the sites as the unit of length. Thus, the time-evolution of the vector $\vec {\phi }=(\phi _0, \phi _1)^{\mathrm{T}}$ is described by

2 $$ \partial _t \vec {\phi }(t) = -D \left( \begin{array}{cc} 1 &{} -1 \\ -1 &{} 1 \end{array}\right) \vec {\phi }(t). $$

Therefore, for a one-dimensional chain composed of $L_x$ sites (see Fig. 1b), the time-evolution of the vector $\vec {\phi }=(\phi _0, \phi _1,\ldots ,\phi _{L_x-1})^{\mathrm{T}}$ is described by

3a $$ \partial _t \vec {\phi }(t) = -\hat{H}\vec {\phi }(t), $$

3b $$ \hat{H} = D\left( \begin{array}{ccccc} 2 &{}-1 &{} 0 &{} \cdots &{} -1 \\ -1 &{} 2 &{} -1 &{} \cdots &{} 0 \\ 0 &{} -1 &{} 2 &{} \cdots &{} 0 \\ \vdots &{} \vdots &{}\vdots &{} \ddots &{} \vdots \\ -1 &{} 0 &{} 0 &{} \cdots &{} 2 \end{array}\right) , $$

which is a discretized form of the diffusion Eq. (1). Here, we have imposed the periodic boundary condition. In Sec. I of Supplemental Material, we derive Eq. (3) for $L_x=3$. Equation (3) bridges the diffusion phenomena and quantum systems; the matrix $\hat{H}$ corresponds to the Hamiltonian of a one-dimensional tight-binding model.

In the continuum limit, Eq. (3) is reduced to Eq. (1). To see this, we diagonalize the matrix $\hat{H}$ and focus on the long-wavelength limit. By applying the Fourier transformation, $\phi _{j_x} = \frac{1}{\sqrt{L_x}} \sum _{k_x} e^{ik_x j_x} \phi _{k_x}$, we obtain eigenvalues as $\epsilon (k_x)=D(2-2\cos k_x )$ with $k_x=2\pi n_x/L_x$ ($n_x=0,1,\ldots ,L_x-1$). For $k_x\sim 0$, we have $\epsilon (k_x)\sim Dk^2_x$, meaning that the time-evolution is described by Eq. (1) in the long-wavelength limit. Here we have used the correspondence $k_x \leftrightarrow -i \partial _x$.

In the above, by discretizing the diffusion equation, we have shown that the diffusive dynamics of classical systems can be described by the tight-binding model of quantum systems [see Eq. (3)]. This result implies that the diffusive systems serve as a new platform of topological physics beyond quantum systems.

## SSH model of the heat conduction equation

**Figure 2:**
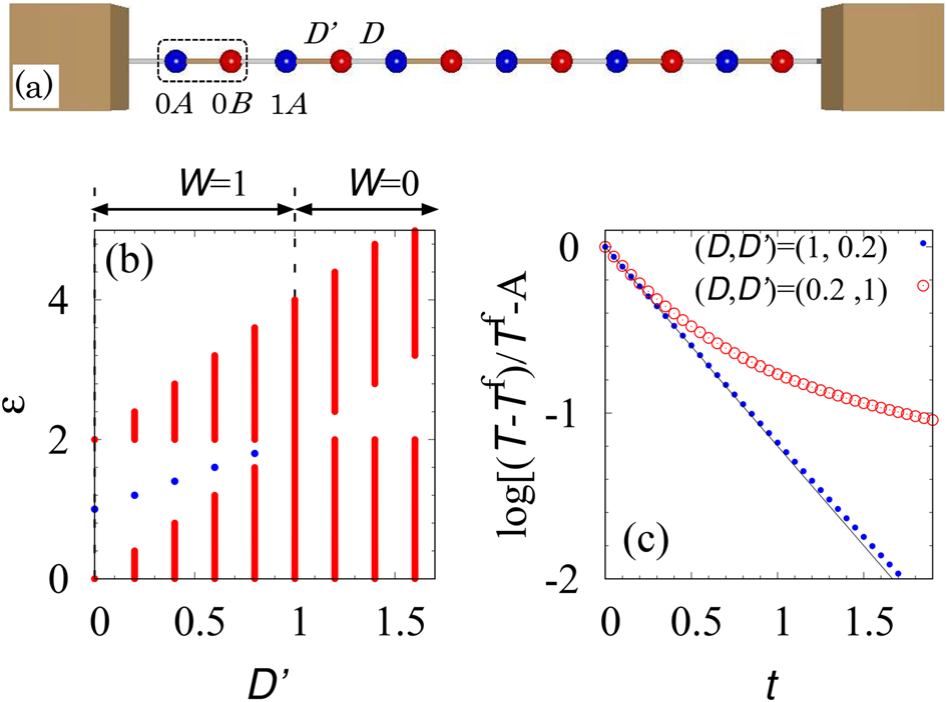
(Color Online). (**a**) Sketch of the model under the fixed boundary condition for $L_x=6$. The sites labeled by $(i_x,\alpha )$ are coupled to the neighboring sites or walls whose coupling strength is denoted by the diffusion coefficient $D'$ (brown) or *D* (gray). We assume that the heat capacity of the wall is sufficiently large, i.e., the wall works as a heat bath. (**b**) Spectrum of $\hat{H}_{\mathrm{SSH}}$ for $D=1$ and $L_x=240$. Here, the fixed boundary condition is imposed. For $0\le D'<1$ the system exhibits the edge states denoted by blue dots because of the bulk topological properties. (**c**) The time-evolution of $\vec {T}_{0A}(t)$ in the case for $(D,D')=(1,0.2)$ [$(D,D')=(0.2,1)$] where the system is topologically nontrivial (trivial). The function $-(D+D')t$ is plotted with a black line. We have subtracted $A=\log [(T_{0A}(t=0)-T^{ \mathrm {f} })/T^{ \mathrm {f} }]$ for comparison. The data are obtained with $T^{ \mathrm {f} }=T_{0A}(t=50)$ and $L_x=240$. We set the initial state as $\vec {T}_{i\alpha }=\delta _{i0}\delta _{\alpha A}$. These data are obtained by imposing Dirichlet-type boundary conditions. Namely, for the spatial direction, we imposed the fixed boundary condition. For time-direction, we imposed the initial condition $T(0)_{i\alpha }=\delta _{i0}\delta _{\alpha A}$. In either case of parameters, the temperature decays monotonically, which is compatible with the boundary condition of time $\vec {T}(\infty )_{i\alpha }=0$.

In order to demonstrate that the diffusive dynamics of classical systems indeed shows topological phenomena we analyze a one-dimensional system with dimerization (see Fig. 2a) which corresponds to the Su-Schrieffer-Heeger (SSH) model^42,43^ of quantum systems. In the rest of this paper, we discuss the discretized version of the heat conduction equation for the sake of concreteness.

Let us consider a one-dimensional system illustrated in Fig. 2a. The temperature at each site is described by the following vector, $$\vec {T}=\left( \begin{array}{ccccc} T_{0A}&T_{0B}&T_{1A}&\cdots&T_{L_x-1B} \end{array} \right) ^{\mathrm{T}}$$. Here, the temperature at each site $T_{i_x\alpha }$ ($\alpha =A,B$) is defined as the difference from the temperature of the wall $T_{\mathrm{w}}$.

In a similar way to derive Eq. (3), we obtain the following equation

4 $$ \partial _t \vec {T}(t) = -\hat{H}_{\mathrm{SSH}}\vec {T}(t), $$

with $\delta :=D'/D>0$. For details of the derivation and the specific form of the matrix $\hat{H}_{\mathrm{SSH}}$, see Sec. IIA of Supplemental Material.

Firstly, let us discuss topological properties in the bulk. Namely, temporarily abandoning the boundary condition illustrated in Fig. 2a, we first impose the periodic boundary condition. In the momentum space, the matrix $\hat{H}_{\mathrm{SSH}}$ is rewritten as

5 $$ \hat{h}_{\mathrm{SSH}}(k_x) = D \left( \begin{array}{cc} 1+\delta &{} \delta +e^{i k_x} \\ \delta +e^{-i k_x} &{} 1+\delta \end{array}\right), $$

with $k_x=2\pi n_x /L_x$ and $n_x=0,1,\ldots , L_x-1$. Here, the corresponding vector denoting the temperature is written as $$\vec {T}(k_x)= \left( \begin{array}{cc} T_A(k_x)&T_B(k_x) \end{array}\right) ^{\mathrm{T}}.$$ The Pauli matrices $\sigma$’s act on the sublattice degrees of freedom [$$\sigma _1=\left( \begin{array}{cc} 0 &{} 1\\ 1 &{} 0 \end{array} \right) $$, $$\sigma _2=\left( \begin{array}{cc} 0 &{} -i\\ i &{} 0 \end{array}\right) $$, and $$\sigma _3=\left( \begin{array}{cc} 1 &{} 0\\ 0 &{} -1 \end{array}\right) $$]. Before analyzing the topological properties, we note that the system shows a gap and preserves the chiral symmetry. Diagonalizing the matrix, we obtain the spectrum $\epsilon _{\pm }(k_x)=D \left[ (1+\delta ) \pm \sqrt{(\delta +\cos k_x)^2+\sin ^2 k_x} \right]$. This result indicates that the spectrum shows a gap for $\delta \ne 1$. The system also preserves the chiral symmetry; $\hat{h}'_{\mathrm{SSH}}:=\hat{h}_{\mathrm{SSH}} -D(1+\delta )\sigma _0$ satisfies $\sigma _3 \hat{h}'_{\mathrm{SSH}} (k_x) \sigma _3=-\hat{h}'_{\mathrm{SSH}}(k_x)$. Here, we note that the shift described by the identity matrix $\sigma _0$ does not affect the eigenvalue problem, meaning that topological properties of the eigenvectors are encoded into $\hat{h}'_{\mathrm{SSH}}$.

Because $\hat{h}'_{\mathrm{SSH}}$ shows the gap and preserves the chiral symmetry, it may possesses the topologically nontrivial properties which are characterized by the winding number:

6 $$ W= -\int ^\pi _{-\pi } \frac{dk_x}{4\pi i} {\mathrm{tr}}[\sigma _3 \hat{h}'^{-1}_{\mathrm{SSH}}(k_x) \partial _{k_x} \hat{h}'_{\mathrm{SSH}}(k_x)]\in {\mathbb {Z}}. $$

The winding number counts how many times the off-diagonal element of $\hat{h}_{\mathrm{SSH}}$ winds around the origin of the complex plane, and thus, it takes an integer^44^. Computing the winding number, we can see that the winding number takes the value one ($W=1$) for $0 \le D' < 1$ while it takes the value zero ($W=0$) for $1 \le D'$.

For one-dimensional quantum systems with chiral symmetry, the winding number predicts the number of the gapless edge modes localized around the edges, which is typical example of the bulk-edge correspondence. We show that the bulk-boundary correspondence can be observed in our classical system. Figure 2b shows the spectrum of $\hat{H}_{\mathrm{SSH}}$ under the fixed boundary condition. This figure indicates that corresponding the winding number $W=1$ ($W=0$), there exists an edge state (no edge state) localized at each edge, which is represented as a blue dot for each value of $D'$. Here, the edge state appears at $\epsilon =D+D'$ because of the term proportional to the identity matrix.

The above results demonstrate that the diffusive dynamics of classical systems exhibit the bulk-edge correspondence which is a unique topological phenomenon. We stress that the edge states appear due to the topological properties in the bulk, which implies the following behaviors as is the case of quantum systems^10^: (1) The edge states survive even in the presence of perturbation (2) A states is localized at the boundary of two SSH models $\delta >1$ and $\delta <1$ which would results in the essentially same behavior as the one shown in Fig. 2c. We note that imposing the free boundary condition breaks the chiral symmetry, which shifts the eigenvalue of the edge state away from $D(1+\delta )$.

## How to experimentally access the edge states

So far, we have shown that the edge states emerge at $\epsilon =D+D'$ because of the topological properties in the bulk. In the following, let us discuss how to experimentally access the edge states.

One possibility is to observe the time-evolution of the temperature at the edge which is considered to decay exponentially $T_{0A}\sim e^{-(D+D')t}$. In Fig. 2c, the time-evolution of the temperature at edge $(i_x,\alpha )=(0,A)$ is plotted. The temperature $T_{0A}$ shows exponential decay for $t\lesssim 2 \tau$ with the half-life $\tau =1/(D+D')=0.83$ for $(D,D')=(1,0.2)$ due to the edge state, while it deviates from the line of the exponential decay around $t=0.5$ which is shorter than the half-life for $(D,D')=(0.2,1)$. The above behaviors should be observed even in the presence of the disorder preserving the chiral symmetry. This is because regardless of the details of the system, a finite value of the winding number predicts edge states localized around the boundary as is the case of quantum systems. Therefore, we conclude that observing the time-evolution allows us to experimentally access the edge states induced by the bulk topological properties. We note that the time-evolution of the temperature at each site has been measured in Ref.^38^ for continuous systems; when the system is composed of aluminum, the half-life $\tau$ is estimated to be $\tau \sim 1\mathrm {ms}$ (for more details, see Sec. IIB of Supplemental Material).

We also consider that at least in principle, the eigenvectors and eigenvalues of the matrix $\hat{H}_{\mathrm{SSH}}$ can be extracted from the experimental data in the following procedure. (1) Prepare a set of initial conditions $\vec {T}^{(\mathrm {i})}(t=0)_{l}$ ($l=0,\ldots ,L_x-1$) which are linearly independent each other; for instance, such initial conditions can be prepared by heating at a site. (2) Observe the temperature $\vec {T}^{(\mathrm {f})}_{l}$ at time $t_0$ for each case of initial condition. Here, these two sets of experimental data satisfy

7 $$ \hat{T}(t_0) = e^{-H_{\mathrm{SSH}} t_0} \hat{T}(0), $$

with $\hat{T}(t_0)=(\vec {T}^{(\mathrm {f})}_{0}, \vec {T}^{(\mathrm {f})}_{1}, \ldots , \vec {T}^{(\mathrm {f})}_{L_x-1})$ and $\hat{T}(0)=(\vec {T}^{(\mathrm {i})}_{0}, \vec {T}^{(\mathrm {i})}_{1}, \ldots , \vec {T}^{(\mathrm {i})}_{L_x-1})$. (3) Diagonalizing $\hat{T}(t_0)[\hat{T}(0)]^{-1}$, which is identical to $e^{-\hat{H}_{\mathrm{SSH}} t_0}$, we obtain the eigenvalues and eigenstates of $\hat{H}_{\mathrm{SSH}}$.

**Figure 3:**
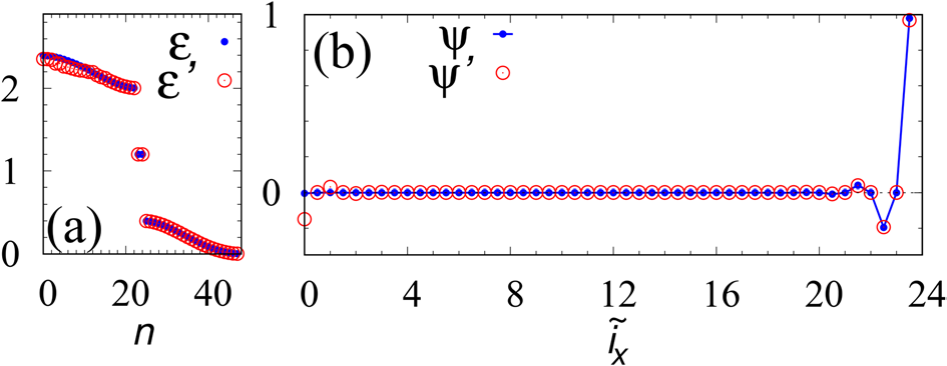
(Color Online). (**a**), (**b**) The eigenvalues (the edge state) obtained from $\hat{H}_{\mathrm{SSH}}$ and $\hat{T}(t_0)[\hat{T}(0)]^{-1}$ for $D=1$, $D'=0.2$, $L_x=24$, and $t_0=17$. The eigenvalues of the matrix $\hat{T}(t_0)[\hat{T}(0)]^{-1}$ are defined as $e^{-\epsilon '_n t_0}$. The set of labels $(i_x,\alpha )$ is represented as $\tilde{i}_x$ as follows: $\tilde{i}_x$ takes $i_x$ [$i_x+0.5$] for $(i_x,A)$ [$(i_x,B)$].

Figure 3a shows eigenvalues of $\hat{T}(t_0)[\hat{T}(0)]^{-1}$. The eigenvalues are obtained with the initial condition $[\vec {T}^{(\mathrm {i})}_l]_{i_x\alpha } =T_{l\alpha }\delta _{li_x}\delta _{{\alpha _l}\alpha }$ with $\alpha _l=A,B$ and $T_{l\alpha }$ taking a random value between 0.5 and 1. (Concerning the temperature, specific choice of the unit does not matter because the ratio of the temperature is discussed throughout this paper.) The eigenvalues $e^{-\epsilon ' t_0}$ almost reproduce the ones of $\hat{H}_{\mathrm{SSH}}$. We note that the deviation for $2.5 \lesssim \epsilon \lesssim 3$ is due to the rounding error; the matrix elements of $\hat{T}(t_0)_{ji}$ exponentially decay. Figure 3b shows the edge state $\psi '$ obtained from the matrix $\hat{T}(t_0)[\hat{T}(0)]^{-1}$ which corresponds to the edge mode. The eigenvector $\psi '$ also is in nice agreement with the edge state $\psi$ of $\hat{H}_{\mathrm{SSH}}$.

## Honeycomb lattice system

**Figure 4:**
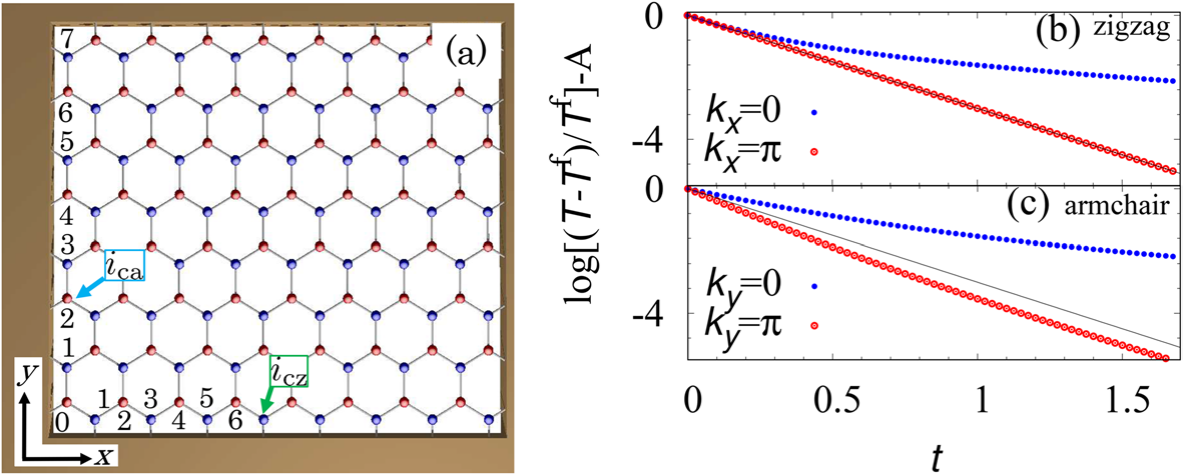
(Color Online). (**a**) Sketch of the honeycomb lattice for $L_x=16$ and $L_y=8$. Here, the fixed boundary condition is imposed both for the *x*- and *y*-directions. The numbers along the *x*- (*y*-) direction represent $i_x=0,1,2,\ldots ,15$ ($i_y=0,1,2,\ldots ,7$). (**b**), (**c**) Time-evolution of temperature $T_{i_{\mathrm{cz}}}$ [$T_{i_{\mathrm{ca}}}$] along a zigzag edge [an armchair edge] for $L_x=L_y=40$ and $D=1$. The subscript $i_{\mathrm{cz}}=(L_x/2-1,0)$ [$i_{\mathrm{ca}}=(0,L_y/2-2)$] specifies the site on the zigzag [armchair] edge. For $L_x=16$ and $L_y=8$, the site specified by $i_{\mathrm{cz}}=(7,0)$ [$i_{\mathrm{ca}}=(0,2)$] is denoted by the green (blue) arrow in panel (**a**). The function $-3Dt$ is plotted with a black line. The temperature $T^{\mathrm{f}}$ is set to $T_{i_{\mathrm{cz}}}(t=50)$ [$T_{i_{\mathrm{ca}}}(t=50)$] for the zigzag (armchair) edge. We have subtracted $A=\log [(T_{i_{\mathrm{cz}}(i_{\mathrm{ca}})}(t=0)-T^{\mathrm{f}})/T^{\mathrm{f}}]$ for comparison.

Topological phenomena of the diffusive dynamics can also be found for two-dimensional systems. To show this, we analyze a honeycomb lattice system illustrated in Fig. 4a. We have supposed that the sites are coupled with the diffusion coefficient *D*. The dynamics of the temperature at each site $\vec {T}$ is described by $\partial _t \vec {T} =-\hat{H}_{\mathrm{honey}} \vec {T}$. As is the case of the SSH model, $\hat{H}_{\mathrm{honey}}$ corresponds to the honeycomb lattice of the tight-binding model; $\hat{H}'_{\mathrm{honey}}:=\hat{H}_{\mathrm{honey}}-3D1\!\mathrm{l}$ preserves the chiral symmetry.

Under the periodic (fixed) boundary condition for the *x*- (*y*-) direction, the system can be regarded as a set of one-dimensional systems aligned along the momentum space $-\pi \le k_x < \pi$. Noting that the one-dimensional system specified by $k_x$ preserves the chiral symmetry, we can compute the winding number; the winding number takes the value one ($W=1$) for $2\pi /3< |k_x| < \pi$, while it takes the value zero ($W=0$) for $0 \le |k_x| < 2\pi /3$. Correspondingly, only for $2\pi /3<|k_x|<\pi$, the edge state appears^13,45^. We note that along the armchair edge, no edge states can be observed. For more details of the spectrum, see Sec. IIIA of Supplemental Material.

The presence or absence of the edge state can affect the diffusive dynamics. Figure 4b,c shows the time-evolution at site $i_{\mathrm{cz}}$ ($i_{\mathrm{az}}$). Figure 4b shows the dynamics obtained for the two cases of the initial condition spatially modulating either $k_x=0$ or $k_x=\pi$. The data of $k_x=\pi$ are obtained by subtracting data obtained with the initial condition $2\vec {T}_{\mathrm{iz1}}$ from the ones obtained with $\vec {T}_{\mathrm{iz2}}$ (For specific form of $\vec {T}_{\mathrm{iz1}}$ and $\vec {T}_{\mathrm{iz2}}$, see Sec. IIIB of Supplemental Material). Figure 4b indicates that at the zigzag edge, the temperature field with $k_x=\pi$ exponentially decays $T_{\mathrm{icz}}\sim e^{-3Dt}$ while the data of the temperature field with $k_x=0$ deviates from $e^{-3Dt}$. The above time-evolution is consistent with the presence of the edge state for $2\pi /3< |k_x| <\pi$ whose eigenvalue is 3*D*. We note that the time-evolution of the armchair edge deviates from $e^{-3Dt}$ for either initial condition (see Fig. 4c).

**Figure 5:**
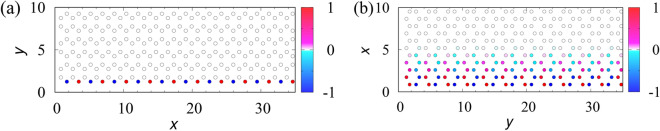
(Color Online). (**a**), (**b**) Color plot of $\vec {T}(t=1)/T_0$ for a zigzag (an armchair) edge. The initial condition is chosen as $\vec {T}_{\mathrm{iz3}}$ ($\vec {T}_{\mathrm{ia3}}$) for data of zigzag (armchair) edges, which allows us to observe the mode with $k_x=\pi$ ($k_y=\pi$) for the zigzag (armchair) edge. For more details of the initial condition, see Fig. 2 and Sec. IIIB of Supplemental Material. The data shown in panel (**a**), (**b**) are obtained under the periodic and fixed (fixed and periodic) boundary conditions along the *x*- and *y*-directions. Here, we have taken $T_0=0.0497$ (0.0292) for data of zigzag (armchair) edges. The data are obtained for $L_x=L_y=40$ and $D=1$.

Furthermore, the edge state at $k_x=\pi$ results in counter intuitive dynamics; for the zigzag edge, the initial state with $k_x=\pi$ cannot diffuse to the bulk (see Fig. 5a) while for the armchair edge, the initial state diffuses to the bulk (see Fig. 5b). This intriguing behavior is due to the complete localization of the edge state with $k_x=\pi$. The above counterintuitive behavior is due to the complete localization of the state around the zigzag edge.

## Summary

In this paper, we have elucidated the topological aspect of the diffusive dynamics, providing a new platform of the bulk-edge correspondence.

Specifically, based on Fick’s law, we have introduced the discretized form of the diffusion equation, bridging the diffusive dynamics of classical systems and a tight-binding model discussed for quantum systems. The correspondence between the classical and quantum systems allows us to discuss the topological phenomena (e.g., the bulk-edge correspondence) for the diffusive dynamics of classical systems; we have numerically elucidated that topological properties characterized by the winding number in the bulk induces the edge states for the one-dimensional system and the honeycomb lattice system. Furthermore, our numerical simulation has revealed a novel diffusion phenomenon for the honeycomb lattice system; at zigzag edges, the temperature field with spatial modulation $k_x=\pi$ cannot diffuse to the bulk.

Our results provide topological insights into diffusion phenomena, indicating the potential existence of diffusion phenomena analog of topological insulators for other symmetry classes and higher-order insulators. Their realization is left as future works to be addressed.

## Supplementary Information

Supplementary Information 1.
