# Supplementary material for: Bulk-edge correspondence of classical diffusion phenomena
Source: Sci Rep. 2021 Jan 13;11:888. doi: 10.1038/s41598-020-80180-w (PMC7806654; doi:10.1038/s41598-020-80180-w)
Supplement: Supplementary file 1 — Supplementary Information 1. [file 41598_2020_80180_MOESM1_ESM.pdf]

Supplemental Materials: Bulk-edge correspondence of classical diffusion phenomena

Tsuneya Yoshida[1] and Yasuhiro Hatsugai[1]

[1]*Department of Physics, University of Tsukuba, Ibaraki 305-8571, Japan*

(Dated: November 6, 2020)

I. DERIVATION OF EQ. (3) FOR $L_x = 3$

Equation (3) can be straightforwardly obtained from Eq. (2). For the sake of concreteness, we consider the case for $L_x = 3$ [see Fig. 1(b)]. In this case, the time-evolution of the vector $\vec{\phi}(t) = (\phi_0, \phi_1, \phi_2)^{\mathrm{T}}$ is specified by

$$\partial_t \vec{\phi}(t) = -D[\begin{pmatrix} 1 & -1 & 0 \\ -1 & 1 & 0 \\ 0 & 0 & 0 \end{pmatrix} + \begin{pmatrix} 0 & 0 & 0 \\ 0 & 1 & -1 \\ 0 & -1 & 1 \end{pmatrix} + \begin{pmatrix} 1 & 0 & -1 \\ 0 & 0 & 0 \\ -1 & 0 & 1 \end{pmatrix}]\vec{\phi}(t). \tag{1}$$

Simplifying the right hand side of the above equaiton, we obtain

$$\partial_t \vec{\phi}(t) = -D \begin{pmatrix} 2 & -1 & -1 \\ -1 & 2 & -1 \\ -1 & -1 & 2 \end{pmatrix} \vec{\phi}(t), \tag{2}$$

which is nothing but Eq. (3) for $L_x = 3$.

II. DETAILS OF THE SSH MODEL

A. Derivation of Eq. (4)

Here, we derive the heat conduction equation, Eq. (4), for the system illustrated in Fig. 2(a).

Firstly, let us start with the case of $D' = 0$. In this case, the isolated site is coupled to the wall. By making use of Fourier's law, the heat flux from site $(i_x, \alpha) = (0, A)$ to the wall is written as

$$\vec{Q}_{0A\to\mathrm{w}} = -D(T_{0A} - T_{\mathrm{w}}), \tag{3}$$

where D denotes the diffusion coefficient. The temperatures at site $(i_x, \alpha) = (0, A)$ and the wall are denoted by T_{0A} and T_{w}, respectively.

Because the heat at each site is rewritten as the temperature with the heat capacity, we have

$$\begin{pmatrix} C_w & 0 \\ 0 & C \end{pmatrix} \partial_t \begin{pmatrix} T_{\mathrm{w}} \\ T_{0A} \end{pmatrix} = -D \begin{pmatrix} 1 & -1 \\ -1 & 1 \end{pmatrix} \begin{pmatrix} T_{\mathrm{w}} \\ T_{0A} \end{pmatrix}, \tag{4}$$

where C_{w} and C denote the heat capacity of the wall and site $(i_x, \alpha) = (0, A)$.

By multiplying the matrix $\begin{pmatrix} C_{\mathrm{w}} & 0 \\ 0 & C \end{pmatrix}^{-1}$ from left, the above equation is rewritten as

$$\partial_t \begin{pmatrix} T_{\mathrm{w}} \\ T_{0A} \end{pmatrix} = -D \begin{pmatrix} 1/C_w & -1/C_w \\ -1/C & 1/C \end{pmatrix} \begin{pmatrix} T_{\mathrm{w}} \\ T_{0A} \end{pmatrix}. \tag{5}$$

When C_{w} is infinitely large, T_{w} becomes independent of time. With this approximation and defining $T_{\mathrm{w}} = 0$, we have

$$\partial_t \begin{pmatrix} 0 \\ T_{0A} \end{pmatrix} = -\frac{D}{C} \begin{pmatrix} 0 & 0 \\ -1 & 1 \end{pmatrix} \begin{pmatrix} 0 \\ T_{0A} \end{pmatrix}. \tag{6}$$

In a similar way, we have the heat conduction equation Eq. (4) for $D' \neq 0$. Namely, the time-evolution of the temperatures

$$\vec{T} = \begin{pmatrix} T_{0A} & T_{0B} & T_{1A} & \cdots & T_{L_x-1A} & T_{L_x-1B} \end{pmatrix}, \tag{7}$$

are given by

$$\partial_t \vec{T}(t) = -\hat{H}_{\mathrm{SSH}} \vec{T}(t), \tag{8a}$$

$$\hat{H}_{\mathrm{SSH}} = D \begin{pmatrix} 1+\delta & -1 & 0 & \cdots & -\delta \\ -1 & 1+\delta & -\delta & \cdots & 0 \\ 0 & -\delta & 1+\delta & \cdots & 0 \\ \vdots & \vdots & \vdots & \ddots & \vdots \\ -\delta & 0 & 0 & \cdots & 1+\delta \end{pmatrix}, \tag{8b}$$

with $\delta := D'/D$. Here, we note that $\hat{H}_{\mathrm{SSH}} - D(1+\delta)\mathbb{1}$ is identical to the Su-Schrieffer-Heeger (SSH) model, the one-dimensional tight-binding model with the dimerization δ.

B. Estimation of the half-life

When the system is composed of aluminum, the half-life τ is estimated to be $\tau \sim 1$ms. This can be seen as follows. Because our discretized equation should reproduce the thermal condition equation in the continuum limit, we can estimate the coefficient D for $D = D'$. Namely, the thermal diffusivity of aluminum is approximately $\lambda_{\mathrm{Al}} \sim 1 \times 10^{-4}\mathrm{m}^2/\mathrm{s}$ which can be estimated from data shown in Ref. 1. Supposing that we can reproduce the continuum results with 10 sites for system whose length is 2mm, we obtain D; $Da^2 = \lambda_{\mathrm{Al}}$ with a denoting the distance of neighboring sites (i.e., $a = 0.2$mm in this case). Therefore, D is approximately, $D = 2.5 \times 10^3\mathrm{s}^{-1}$, which results in $\tau \sim 1$ms with $\tau \sim 1/D$.

III. DETAILS OF THE HONEYCOMB LATTICE MODEL

A. Spectrum of the honeycomb lattice model

The spectrum of honeycomb lattice model is plotted in Fig. 1. Figure 1(a) shows the spectrum under the periodic and fixed boundary conditions along the x- and y-directions. In this case, applying the Fourier transformation along the x-direction, we can map the two-dimensional system to the one-dimensional system $H_{\mathrm{honey}}(k_x)$ parameterized by k_x. As is the case of the SSH model, $H_{\mathrm{honey}}(k_x)$ preserves the chiral symmetry up to the term proportional to the identity matrix, which allows us to compute the winding number for each value of k_x.

In the case of the zigzag edge, the winding number takes $W = 1$ for $2\pi/3 < k_x < \pi$, inducing the edge modes at $\epsilon = 3D$ [see Fig. 1(a)].

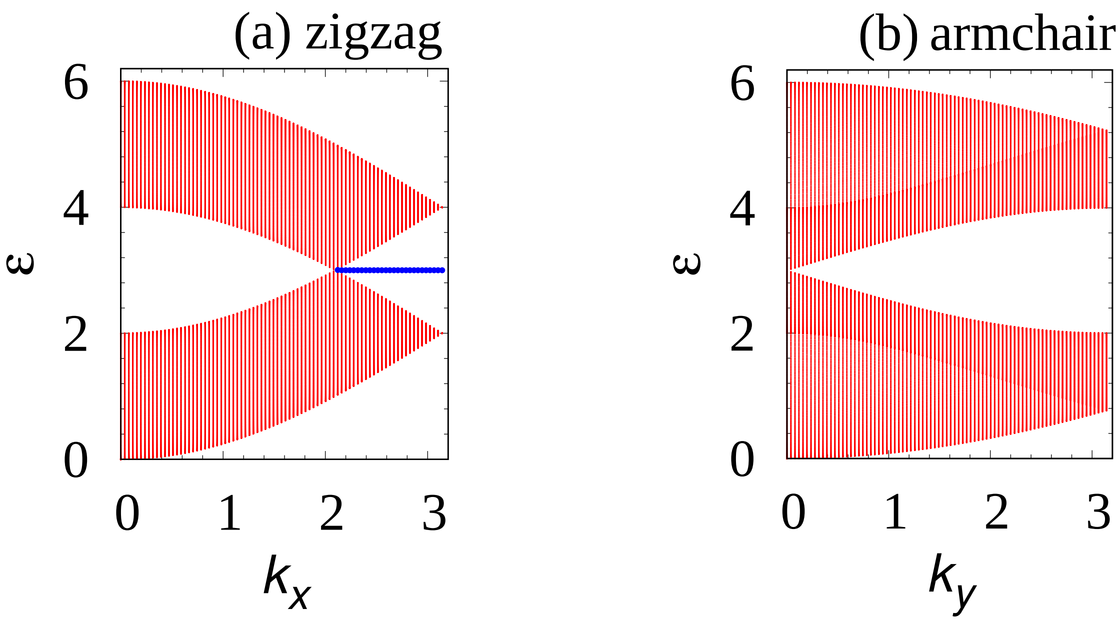

FIG. 1. (Color Online). (a)[(b)]: The eigenvalues for the honeycomb lattice system with zigzag (armchair) edges. For system with zigzag edges, we can find the edge states denoted by blue dots for $2\pi/3 < k_x < \pi$. We note that the spectrum is symmetric about $k_{x(y)} = 0$. The spectrum for zigzag edges (armchair edges) are obtained by imposing the periodic and the fixed (fixed and the periodic) boundary conditions for the x- and y-directions, respectively. These data are obtained for $D = 1$. We suppose that 240 unit cells are aligned along the direction where the fixed boundary condition is imposed.

In the case of the zigzag edge, the winding number is always zero, and thus, no edge state is observed at $\epsilon = 3D$ [see Fig. 1(b)].

B. Initial conditions

In this section we explain the details of the initial conditions which we chose to obtain Figs. 4(b), 4(c), and 5.

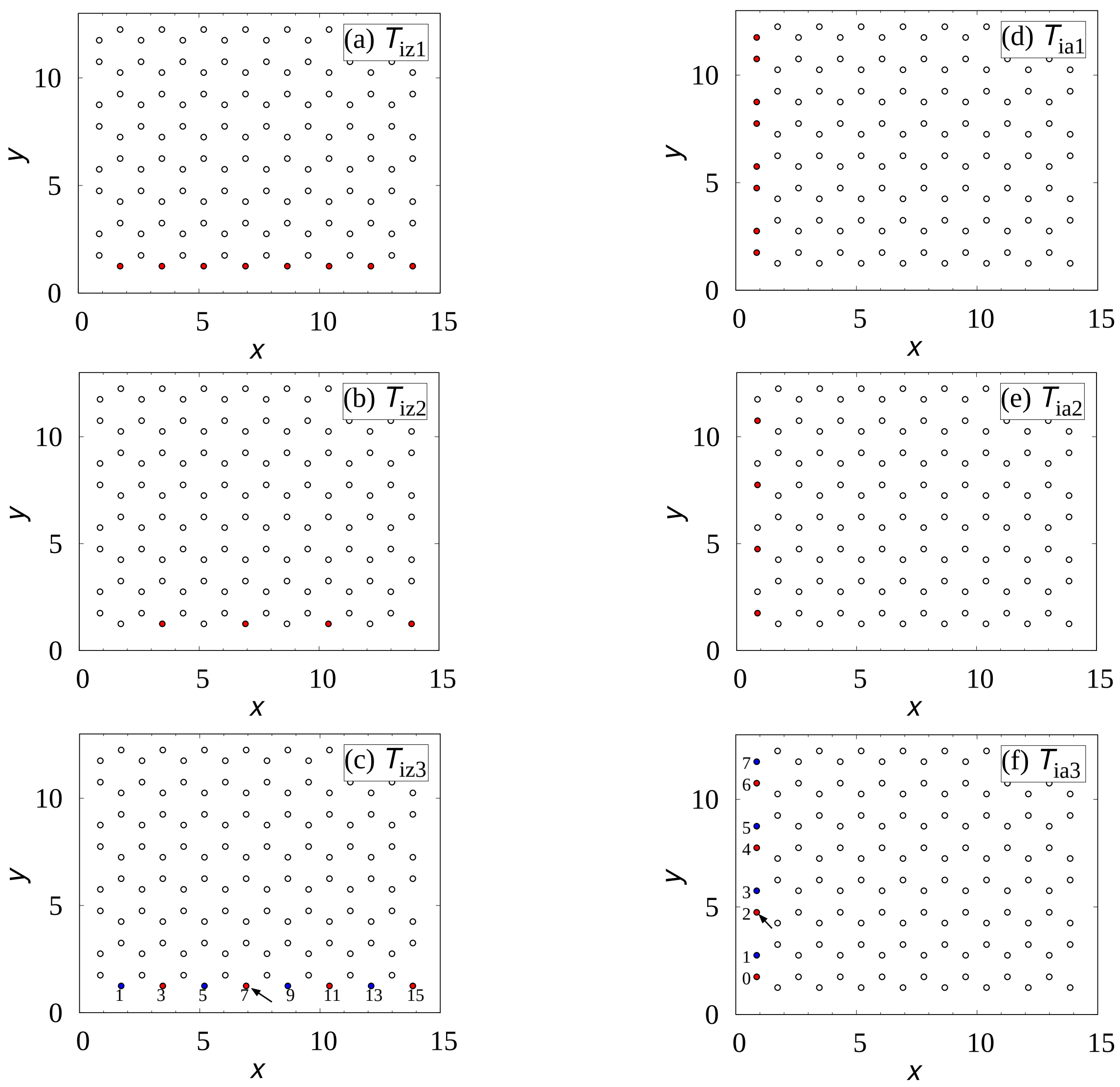

FIG. 2. (Color Online). Color plot of vectors $\vec{T}$'s describing initial conditions for $L_x = 16$ and $L_y = 8$. The red, white, and blue dots denote the points where the temperature is 1, 0, and -1, respectively. In panel (c) [(f)], the site specified by $i_{\rm cz} = (L_x/2-1, 0)$ [$i_{\rm ca} = (0, L_y/2-2)$] is denoted by the arrow. The sites labeled by $i_x = 0, 1, \cdots, L_x - 1$ ($i_x = 0, 1, \cdots, L_y - 1$) as shown in panel (c) [(f)].

Figure 2 shows vectors defining the initial conditions. The data shown in Fig. 4(b) are obtained by simulating the dynamics for two cases of the initial condition: $\vec{T}_{\rm iz1}$ and $2\vec{T}_{\rm iz2}$. The data denoted by $k_x = 0$ are obtained with the initial condition $\vec{T}_{\rm iz1}$. The data denoted by $k_x = \pi$ are obtained by subtracting the data with the initial condition $2\vec{T}_{\rm iz2}$ from the ones with $\vec{T}_{\rm iz1}$. We note that the data labeled by $k_x = \pi$ are identical to the ones with the initial condition $\vec{T}_{\rm iz3} = \vec{T}_{\rm iz1} - 2\vec{T}_{\rm iz2}$ because the diffusion equation is the linear equation. The data shown in Fig. 4(c) are obtained by simulating the dynamics for two cases of the initial condition: $\vec{T}_{\rm ia1}$ or $2\vec{T}_{\rm ia2}$. Namely, the data denoted by $k_y = 0$ ($k_y = 0$) are obtained with the initial condition $\vec{T}_{\rm ia1}$ ($\vec{T}_{\rm ia3} = \vec{T}_{\rm ia1} - 2\vec{T}_{\rm ia2}$).

Figures 5(a) and 5(b) are obtained by simulating the time-evolution with the initial condition $\vec{T}_{\rm iz3}$ ($\vec{T}_{\rm ia3}$).

[1] Ogi, H. *et al.* Thermal mode spectroscopy for thermal diffusivity of millimeter-size solids. *Phys. Rev. Lett.* **117**, 195901 (2016).
